# Reduced Surface Recombination in Extended-Perimeter LEDs toward Electroluminescent Cooling

**DOI:** 10.1021/acsaelm.3c01816

**Published:** 2024-02-13

**Authors:** Luc M. van der Krabben, Natasha Gruginskie, Maarten van Eerden, Jasper van Gastel, Peter Mulder, Gerard J. Bauhuis, Dinar Khusyainov, Dima Afanasiev, Elias Vlieg, John J. Schermer

**Affiliations:** Institute for Molecules and Materials, Radboud University, 6525 AJ Nijmegen, The Netherlands

**Keywords:** electroluminescent cooling (ELC), micro-LEDs (light-emitting diodes), III−V semiconductors, current spreading, perimeter recombination, surface passivation

## Abstract

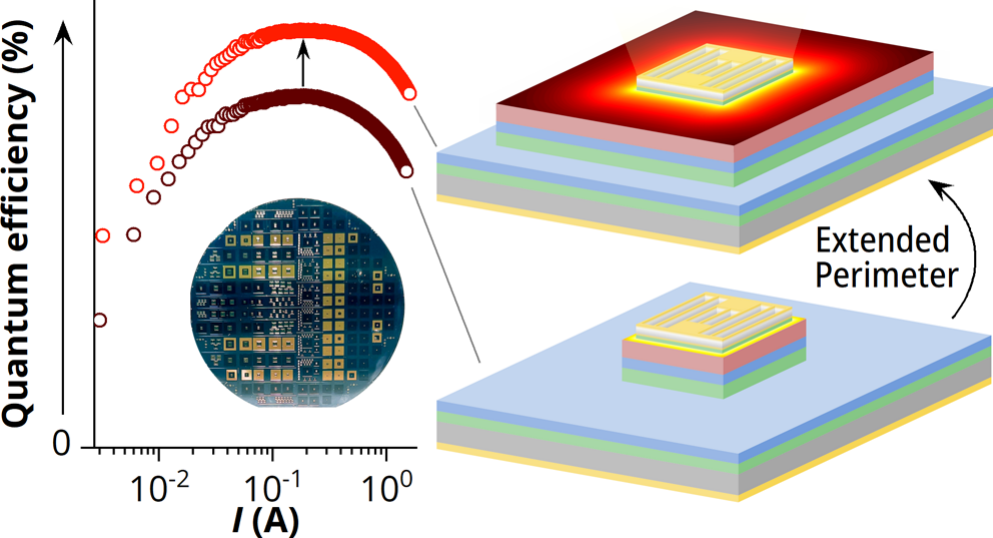

III–V semiconductor light-emitting diodes (LEDs) are a promising candidate for demonstrating electroluminescent cooling. However, exceptionally high internal quantum efficiency designs are paramount to achieving this goal. A significant loss mechanism preventing unity internal quantum efficiency in GaAs-based devices is nonradiative surface recombination at the perimeter sidewall. To address this issue, an unconventional LED design is presented, in which the distance from the central current injection area to the device’s perimeter is extended while maintaining a constant front contact grid size. This approach effectively moves the perimeter beyond the lateral spread of current at an operating current density of 10^1^–10^2^ A/cm^2^. In p–i–n GaAs/InGaP double heterojunction LEDs fabricated with varying sizes and perimeter extensions, a 19% relative increase in external quantum efficiency is achieved by extending the perimeter-to-contact distance from 25 to 250 μm for a front contact grid size of 450 × 450 μm^2^. Utilizing an in-house developed Photon Dynamics model, the corresponding relative increase in internal quantum efficiency is estimated to be 5%. These results are ascribed to a significant reduction in perimeter recombination due to a lower perimeter-to-surface area (P/A) ratio. However, in contrast to lowering the P/A ratio by increasing the front contact grid size of LEDs, the present method enables these improvements without affecting the required maximum current density in the microscopic active LED area under the front contact grid. These findings aid in the advancement of electroluminescent cooling in LEDs and could prove useful in other dedicated semiconductor devices where perimeter recombination is limiting.

## I Introduction

Electroluminescent cooling (ELC) occurs when the amount of optical energy produced by electroluminescence is greater than the injected electrical energy.^1^ To fulfill the first law of thermodynamics, the difference is supplied by the absorption of thermal energy from the crystal lattice, i.e., cooling. The theoretical foundation for this process was laid already in the 1960s, when it was derived that an electroluminescent material should in principle be capable of continuous and reversible conversion between electrical, thermal, and optical energy.^2−6^ However, since the invention of the now-ubiquitous III–V semiconductor light-emitting diode (LED), the practical demonstration of ELC has been obstructed by insufficient material quality and the drive of industry toward maximizing the absolute optical output power of visible LEDs rather than the output/input ratio.^7^ For a full overview of all challenges and practical limitations concerning ELC, Sadi et al. provide a detailed perspective.^1^

In short, to realize a measurable temperature reduction by ELC in LEDs, the LED is required to operate at a wall-plug efficiency (WPE), defined as the ratio of total optical output power to the input electrical power, exceeding unity.^1^ Only then is the LED able to radiate out more power than it receives, indicating its entrance into the ELC regime. The WPE is given by
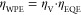
1

The voltage efficiency η_V_ describes how much electrical energy *qV*_b_ is required to generate a photon with energy *hν*:
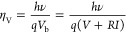
2where *V*_b_ is the applied external LED bias, *V* is the effective internal LED bias, *R* is the sum of all parasitic series resistances, and *I* is the LED current. For the WPE to exceed unity, the voltage efficiency η_*V*_ has to be larger than 1, which implies that the applied voltage should be lower than the photon energy, dictated by the band gap energy (*E*_g_) of the emitting material (1.42 eV for GaAs). For practical cooling powers, however, a high LED current is required and there is hence a limit to the reduction of *qV*_b_.^8^ Therefore, the external quantum efficiency (EQE) η_EQE_, a measurable quantity defined as the ratio of extracted photons to injected charge carriers, should additionally approach unity in the approximate regime .^1^ The EQE follows from three successive processes: charge carrier injection, carrier-to-photon conversion, and photon extraction, each given by its own efficiency:
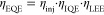
3

The injection efficiency η_inj_ is typically ∼1 for III–V double heterojunction (DH) structures at practical operating biases.^1^ This leaves the challenge to approach unity for both the carrier to photon conversion efficiency η_*IQE*_ or internal quantum efficiency (IQE) and the light extraction efficiency (LEE) η_LEE_, which is the fraction of internally generated photons that eventually escapes the LED, either directly or through the process of photon recycling and re-emission.^9^

The LEE is limited by both loss to the growth substrate and light confinement due to the large refractive index of typical semiconductors, resulting in a small critical angle for total internal reflection of photons crossing the semiconductor-to-air interface.^10,11^ Strategies to address this involve high-refractive index domes on the front and textured mirrors on the back of thin-film LEDs.^1,12,13^ In contrast to these studies, the present work focuses on another necessary step toward the demonstration of ELC, i.e., the optimization of the IQE.

The IQE can be described as the ratio of the radiative recombination rate to all of the recombination rates. This is governed by the competition between first-, second-, and third-order recombination processes. Respectively, these are nonradiative defect-related Shockley–Read–Hall (SRH) recombination, radiative recombination, and Auger recombination.^7,14^ Any parasitic recombination process that is nonradiative needs to be avoided, as this implies a definitive loss in IQE and consequently in cooling power. Nonradiative recombination processes additionally involve heat production, which directly competes with cooling. Moreover, the high current densities (∼10^1^–10^2^ A/cm^2^) necessary to achieve the required cooling power for demonstrating ELC are more easily achieved with small device dimensions,^15^ but this concomitantly increases the sidewall perimeter-to-surface area (P/A) ratio of the LED. Therefore, nonradiative surface recombination at the perimeter sidewall becomes one of the most important loss mechanisms limiting the IQE.^7,10,11^ Over the past few decades, various strategies have been developed to combat perimeter recombination. Examples include chalcogenide-based wet-chemical passivation,^16−18^ field-effect dielectric passivation,^19,20^ wet-chemical or plasma nitridation,^21,22^ and epitaxial regrowth.^23^ However, all of these methods require complex processing techniques or nonstandard equipment and suffer from poor longevity or incomplete passivation.

**Figure 1:**
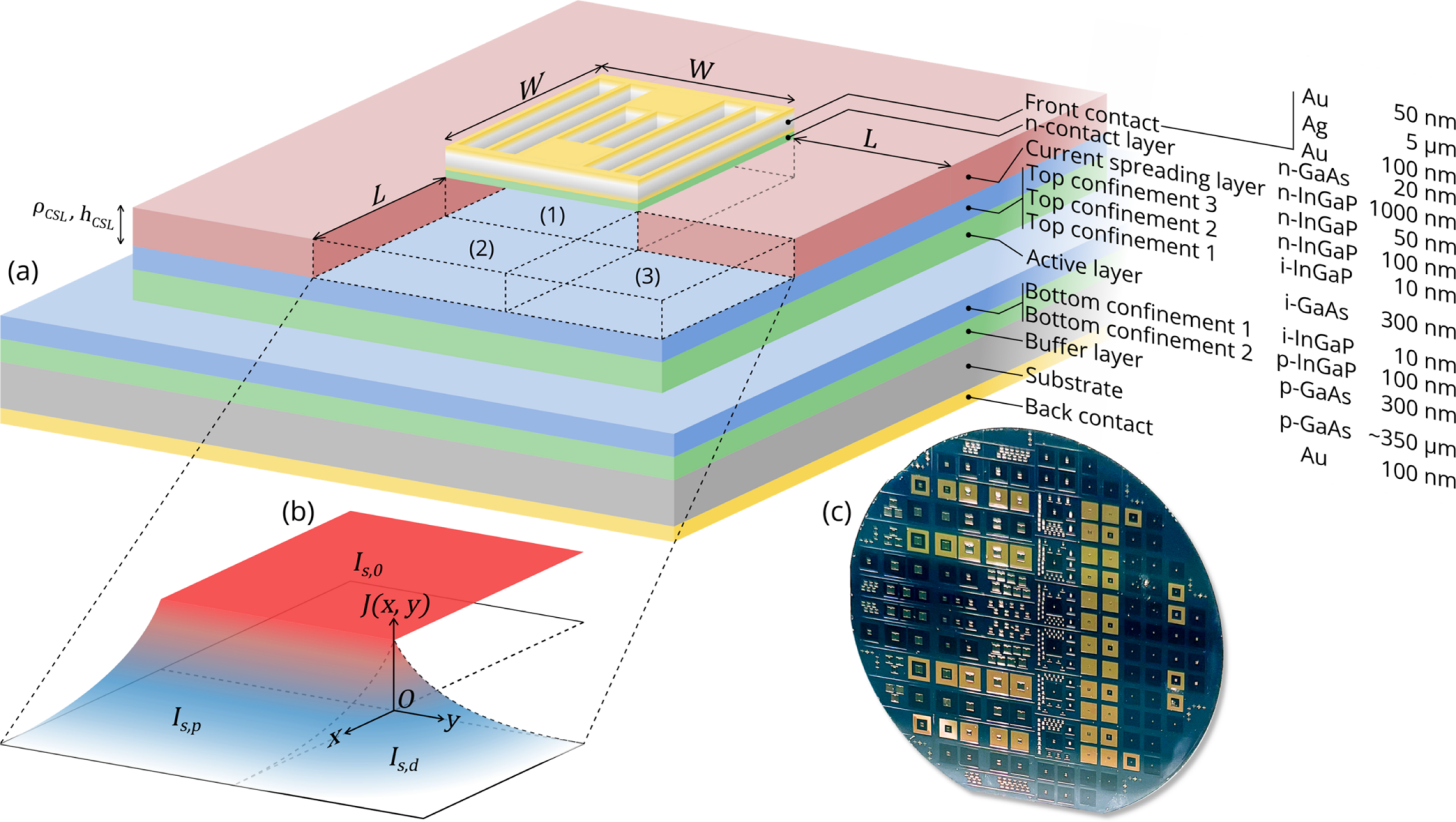
(a) Schematic representation of the fabricated LEDs with a front contact grid width *W* and perimeter extension *L*. *h*_CSL_ and ρ_CSL_ represent the thickness and resistivity of the current spreading layer (CSL), respectively. Note that in the schematic, the CSL is partly cut away to reveal the 3 different current spreading regions on the top confinement layer for which the local current density after spreading in the CSL is being considered in this study. (b) Schematic of the local current density distribution *J*(*x*,*y*) in areas 1, 2, and 3 in (a) at the CSL-top confinement layer interface (i.e., after the injected current has spread from the grid through the CSL). *I*_s,0_, *I*_s,p_, and *I*_s,d_ represent the area integrals of *J*(*x*,*y*) in each of the 3 different areas, showing how current is distributed over the extended perimeter and the *W* × *W* grid width area. The schematics are not to scale. (c) Photograph of a fully processed 2″ wafer with numerous duplicates of GaAs/InGaP LEDs with varying *W* and *L*.

In this study, we report on an easy-to-implement method to circumvent these common problems faced by traditional passivation strategies. Building upon the theory of current spreading, we propose an unconventional design, where the distance between the central carrier injection area and the perimeter is simply extended, such that it is longer than the lateral current spreading length (Figure 1), effectively preventing charge carriers from reaching the perimeter in the first place.^7^ This could significantly reduce perimeter recombination while still enabling high current densities in the small active LED area under the front contact grid.

In this way, extended-perimeter LEDs present a simple approach to perimeter passivation, facilitating direct progress toward demonstrating ELC proof-of-principle devices. To implement this approach, we first developed a model for current spreading to determine the lateral current spreading length as a function of injection current density. Next, a large number of GaAs/InGaP p–i–n double heterojunction (DH) LEDs with varying sizes of front contact grid and perimeter extension (see Figure 1a) are manufactured on the same wafer to demonstrate the influence of the P/A ratio and the concomitant decrease in perimeter recombination. By analyzing the LEDs as they are on the growth substrate, potential variations in LEE that might hinder the direct mutual comparison of the different geometries are avoided, i.e., no individual processing of the devices is carried out to improve the LEE by, for example, the application of domes or local texturing. This results in a low but constant LEE over all of the devices investigated in this study. Consequently, the measured EQE values in this study are low compared to those reported in the literature, where light extraction methods are used to boost the low IQE.^24,25^ Moreover, the choice for GaAs as the emitting material stems from the low Auger recombination and low heat penalty associated with a nonradiative recombination event, compared to lower and higher band gap semiconductor materials, respectively.^9^ Finally, a Photon Dynamics model developed by van Eerden et al. is used to quantify the improvement in IQE.^26^

## II Methods

The p–i–n GaAs/InGaP DH LEDs, as shown in Figure 1a, were grown by using metal–organic chemical vapor deposition (MOCVD) in an Aixtron 200 system at 20 mbar. The epilayers were grown on a p-type doped 2″ (100) GaAs substrate, 2° off to (110) orientation. Zn was used as the p-type dopant and Si as the n-type dopant except for the n-GaAs contact layer, which was highly doped with Te. The active layer, consisting of intrinsic GaAs, was surrounded by p- and n-type InGaP confinement layers with increasingly higher dopant concentration. To verify the effect of the current spreading layer, a second wafer was grown without this layer but otherwise identical epilayer structure to Figure 1a.

After MOCVD growth, the front contact grid of about 5 μm thickness (see Figure 1a), which is needed for dealing with high current densities, was defined with standard photolithography and electron-beam evaporation of gold and silver. The n-GaAs contact layer was removed between the front contact grid using a [1:1:10 v/v] 1 M NaOH/H_2_O_2_/H_2_O etch. Subsequently, the mesa was photolithographically defined and etched with [100:1:200 v/v] HBr/Br_2_/H_2_O up to the bottom confinement layers. For the front contact grid and mesa definition of the LEDs, a 2″ mask was used with different front contact grid widths (*W* = 150–950 μm) and perimeter extensions (*L* = 25–1000 μm), see Figure 1c. Finally, 100 nm of Au was evaporated on the back of the p-type doped substrate to provide a back contact. Chips with different sets of LEDs were cleaved from the wafer and bonded to a PCB using silver paste for the back contacts and 25-μm-diameter 1% SiAl wire bonds for the front contacts.

The devices were then characterized using electroluminescence (EL) imaging and confocal scanning microscopy, where EL was generated by applying a set current using an Aim-TTi PL601 Power Supply. The EL images, intended as a qualitative overview with lower resolution and sensitivity, were taken with a Daheng Imaging MER-2000-19U3M camera with a 1 in. CMOS sensor and a 25 mm f/2.8 objective. For confocal EL scanning microscopy, which provides quantitative data with high resolution and sensitivity, a WITec alpha300S scanning confocal microscope on a piezo-driven scan stage was used in combination with a Zeiss EC Epiplan objective with 20× magnification and 0.4 numerical aperture. The EL signal was collected with a photomultiplier tube. The current–voltage (*J*–*V*) characteristics of the LEDs in the dark were acquired with a Keithley 2601B System SourceMeter on a temperature-controlled table set to 25 °C. For each combination of *W* and *L*, typically 2–5 LEDs were measured to obtain an average *J*–*V* curve with standard deviation. The LED EQE was measured by acquiring EL spectra as a function of the injection current. An Avantes Starline AvaSpec-ULS2048CL-EVO-RS spectrometer with a 100 μm slit size connected to an integrating sphere was used to acquire absolute EL spectra with Avasoft 8.0 software. The injection current was supplied by a Keithley 2460 SourceMeter. The EQE values presented in this study, again averaged over multiple identical LEDs, are corrected for measurement distance to the integrating sphere but not for grid coverage. As only the EQE is a directly measurable quantity and the IQE is not, an in-house developed Photon Dynamics model^26^ is applied to estimate the IQE of the best performing extended-perimeter LEDs produced in this study, in comparison to that of their regular tight perimeter counterparts. A detailed description of this model is given in the Supporting Information.

## III Results and Discussion

### III.I Current Spreading in Extended-Perimeter LEDs

**Figure 2:**
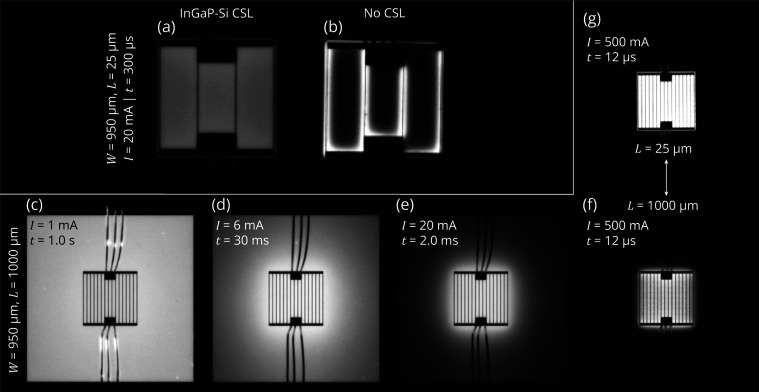
EL images of the GaAs/InGaP LEDs. (a, b) LEDs with wide gridline spacing, with (a) and without (b) a 1000 nm thick Si-doped InGaP CSL at equal input current *I* and exposure time *t*. (c–f) Increasing input currents in extended-perimeter LEDs (*W* = 950 μm and *L* = 1000 μm) with the CSL. Note the decreasing exposure time with an increasing input current. (g) LED with identical front contact grid width *W* and measurement conditions as (f) but with its perimeter close to the front contact grid (*L* = 25 μm).

The importance of adding a current spreading layer (CSL) in the epilayer structure of Figure 1a is qualitatively demonstrated in Figure 2a,b, where LEDs with and without CSL are compared with an exaggerated spacing between the gridlines. Imperfect current spreading is the result of lateral resistance in the semiconductor layers.^7^ This causes significant current crowding in the LED without CSL (Figure 2b), such that most of the EL is emitted underneath the front contact grid. A CSL, on the other hand, ensures homogeneous current spreading throughout the entire *W* × *W* grid width area (Figure 2a). At the anticipated operating current densities (∼10^1^–10^2^ A/cm^2^), a CSL would even be required when the spacing between gridlines is decreased as the extent of lateral spreading of current decreases with increasing input current. Therefore, the LEDs discussed in the remainder of this work will contain a CSL, as in Figure 1a, to ensure homogeneous current spreading throughout the *W* × *W* grid width area.

Building upon the current spreading effect, Figure 2c–f qualitatively demonstrates the concept of extending the contact-to-perimeter distance farther than the current spreading length. With increasingly higher injection currents, the EL in extended-perimeter LEDs (Figure 1) transitions from a very homogeneous distribution over the entire mesa area to emission only close to the front contact grid. However, even at 500 mA, the extended-perimeter LED (*L* = 1000 μm, Figure 2f) still exhibits a gradual transition from light to dark, i.e., from high current density to virtually zero current density. This is in stark contrast to the tight perimeter (*L* = 25 μm, Figure 2g), where charge carriers that otherwise would spread farther than 25 μm reach the perimeter and have a high probability to recombine nonradiatively there.^7^ The area where light is visibly emitted is only slightly larger in the extended-perimeter LED compared with the tight perimeter LED.

**Figure 3:**
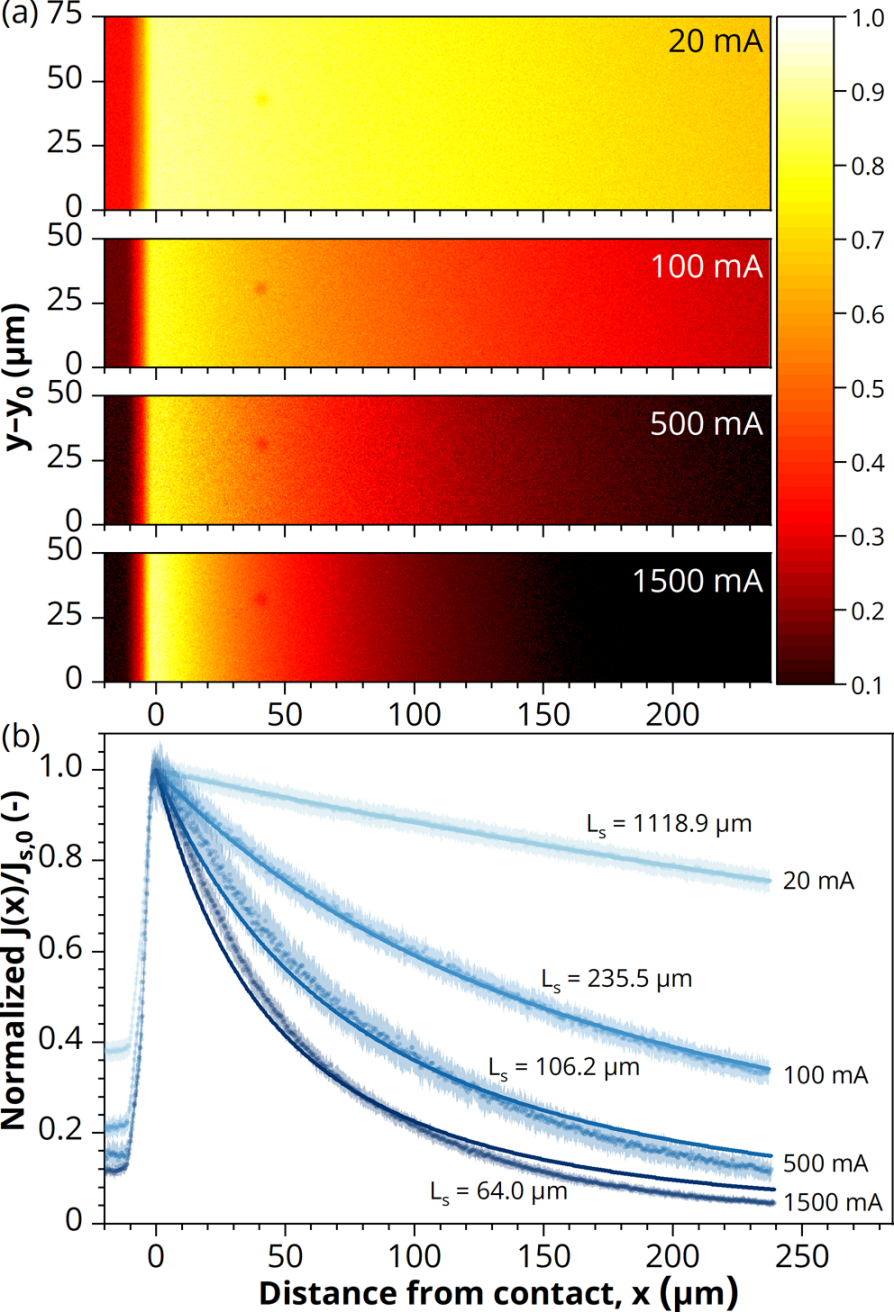
(a) Confocal EL scanning microscopy photon count maps (averaged from 3 maps, normalized on the maximum intensity at *x* = 0 μm) of a GaAs/InGaP LED (*W* = 950 μm and *L* = 1000 μm) with a CSL and input currents of 20, 100, 500, and 1500 mA. The images were taken from the central region of the front contact edge, just aside from the contact pads for wire bonding (i.e., *y*_0_ ≈ −0.3 W). The edge of the broad front contact busbar is seen on the left. The position with the highest EL intensity is set to *x* = 0, such that *x* represents the distance from the edge of the front contact. (b) Normalized current distribution *J*(*x*)/*J*_s,0_ as a function of *x*, as deduced from the EL photon count maps in (a) by averaging over the values along the *y*-direction (the data points represent the average value, and the shaded region represents the standard deviation). Solid lines represent the best fit of eq 4 to the data, resulting in the indicated *L*_s_ values.

To improve the spatial resolution, confocal scanning microscopy is employed on the LED EL. This approach additionally enables us to obtain quantitative values on the current distribution for determining the minimal perimeter-to-contact distance to prevent nonradiative recombination. Figure 3 depicts the drop-off of EL photon count as a function of distance from the edge of the LED front contact grid for increasing injection currents. In order to quantify current spreading, we introduce the current spreading length *L*_s_ using the 1-D current distribution relation for stripe-shaped contacts:^27,28^
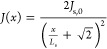
4Here, *L*_s_ is defined as the position *x* from the edge of the front contact grid, at which the current density *J*(*x*) has dropped to approximately 1/3 of its original value underneath the front contact grid, *J*_s,0_ (*x* = *L*_s_ in eq 4). *L*_s_ can be calculated using^28^
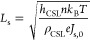
5where *h*_CSL_ and ρ_CSL_ are the thickness and resistivity of the CSL, respectively (see Figure 1a), *n* is the diode ideality factor (∼2), and *k*_B_, *T*, and *e* are the Boltzmann constant, temperature, and elementary charge, respectively. *J*_s,0_ is the current density through the junction underneath the *W* × *W* area after spreading through the CSL. Note that this is distinct from the current density *J*_mesa_ (the input current divided by the total device mesa area, *I*_input_/(*W* + 2*L*)^2^), as well as from *J*_grid_ (the input current before spreading divided by the *W* × *W* grid width area, *I*_input_/*W*^2^). We are interested in finding an expression for *J*_s,0_ since it could be used to determine the current density in extended-perimeter LEDs, as explained at the end of this section. It is assumed that *J*_s,0_ is homogeneous (constant) underneath the *W* × *W* grid width area, which is a reasonable assumption for the LEDs with a CSL (see Figures 2 and S3). Moreover, this description for current spreading assumes that the electrical conductivity of the substrate is infinite, such that the potential is uniform on the p-type side of the junction and that *h*_CSL_ ≪ *L*_s_.^27,28^ The former assumption applies to the thick p-type doped substrate in our epilayer structure (Figure 1a), while the latter assumption is valid only at relatively low input currents. This is indeed observed in Figure 3b, where the fit of eq 4 to the normalized *J*(*x*)/*J*_s,0_, as deduced from the confocal EL scanning microscopy maps (Figure 3a), increasingly deviates from the measurements with a decreasing current spreading length (higher current). More impartially, however, the 1-D current distribution for stripe-shaped contacts does not take into account that current spreads to the corners (area 3 in Figure 1a) as well, which effectively lowers the experimentally determined current density with respect to the linear-stripe case with increasing distances from the front contact grid.

**Figure 4:**
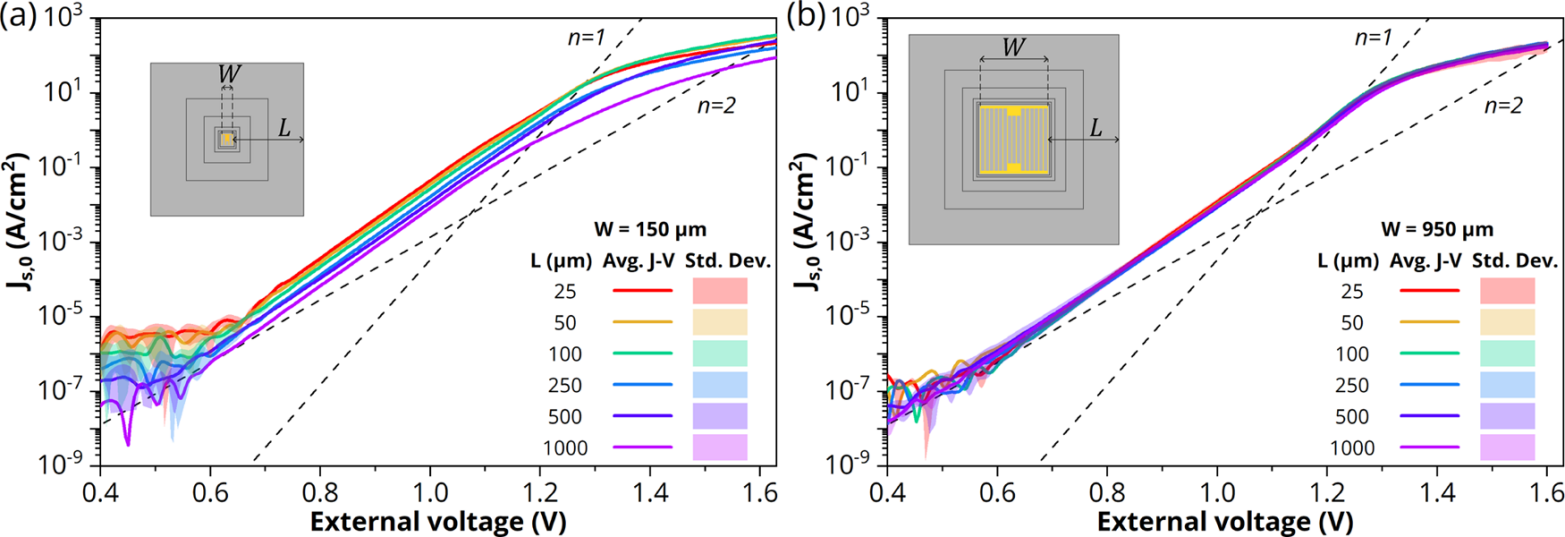
Average current density–voltage (*J*_s,0_–*V*) characteristics in forward bias of GaAs/InGaP LEDs with a CSL. The perimeter extension *L* is varied between 25 and 1000 μm for a front contact grid width of *W* = 150 μm (a) and *W* = 950 μm (b). Inset in panels (a) and (b) are schematics showing the front contact grid width *W* and varying perimeter extension *L*, drawn to scale. In both (a) and (b), the dashed lines represent the single-diode equation *J* = *J*_0*n*_ exp(*qV*/*nk*_B_*T*) with ideality factor *n* = 1 (*J*_01_ = 5.5 × 10^–21^ A/cm^2^) and *n* = 2 (*J*_02_ = 5.5 × 10^–12^ A/cm^2^).

To account for the current leaking away in the corners of the square-shaped geometry, the 1-D theory of current spreading can be expanded to the two-dimensional case of extended-perimeter LEDs. By doing so, it becomes possible to determine *J*_s,0_ and predict *L*_s_ for any input current, current spreading layer, or mesa design. After spreading in the CSL, the input current *I*_input_ is distributed over the *W* × *W* grid width area (*I*_s,0_, area 1 in Figure 1a) and the extended perimeter area, as shown in Figure 1b. We divide the latter into two types of regions: 4 identical spreading regions perpendicular to the side edges of the grid with size *W* × *L* and current *I*_s,p_ (area 2 in Figure 1a) and 4 identical spreading regions diagonal from the corners of the grid with size *L* × *L* and current *I*_s,d_ (area 3 in Figure 1a). The current distribution can therefore be expressed as^29^
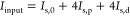
6

To describe the drop in current density with distance from the front contact grid for both the perpendicular and diagonal regions shown in Figure 1b and their 3 equivalents, eq 4 can be approximated to the two-dimensional case in a simple first-order approach:
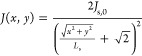
7

To find descriptions for *I*_s,p_ and *I*_s,d_, we have to integrate eq 7 over their respective areas (utilizing the coordinate system as defined in Figure 1b) as shown in eqs 8 and 9.^30^ Inserting eqs 8 and 9 into eq 6 and using the assumption that *J*_s,0_ = *I*_s,0_/*W*^2^ and *J*_input_ = *I*_input_/*W*^2^ represents the current density in the *W* × *W* grid width area before spreading in the CSL, yields eq 10 after some rewriting. Here, the first integral has an exact solution, but the second integral requires a numerical calculation. It becomes clear from eqs 5 and 10 that both *J*_s,0_ and *L*_s_ are dependent on each other and therefore require iterative calculation. This yields the theoretical current spreading lengths, as shown in Table 1.
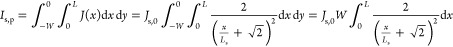
8
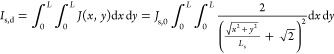
9
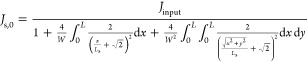
10

**Table 1:** Current Densities (*J*_mesa_, *J*_grid_ and *J*_s,0_) and Current Spreading Length (*L*_s_) in Extended-Perimeter LEDs (*W* = 950 μm and *L* = 1000 μm), with a CSL at Varying Input Currents (*I*_input_)

*I*_input_ (mA)	*J*_mesa_ (A/cm^2^) (=*I*_input_/(*W* + 2*L*)^2^)	*J*_grid_ (A/cm^2^) (=*I*_input_/*W*^2^)	*J*_s,0_ (A/cm^2^)^a^ (eqs 5 and 10)	*L*_s_ (μm)^a^ (eqs 5 and 10)
20	0.23	2.2	0.43	872
100	1.1	11	3.8	295
500	5.7	55	32	101
1500	17	166	123	51.8

a *J*_s,0_ and *L*_s_ are iteratively calculated with eqs 5 and 10 (*T* = 300 K, *n* = 2, *h*_CSL_ = 1150 nm, ρ_CSL_ = 1.81 × 10^–5^ Ωm (measured in-house)).

This first-order approach for describing current spreading can aid in the interpretation of the current–voltage characteristics of extended-perimeter LEDs. In particular, the fact that the mesa area is only partially utilized (see, for example, Figure 2f) raises the question of what the actual current density through the junction in the active LED area under the front contact grid is. As previously described, this is neither equal to *J*_mesa_ nor to *J*_grid_, but a value in-between, depending on the extent of current spreading. Based on our model, *J*_*s*,0_ describes the current density through the junction underneath the *W* × *W* grid width area after spreading through the CSL and therefore takes into account that a certain part of the total input current has spread to the extended perimeter (see Table 1 for a comparison between the different current densities). Specifically, at infinitesimal current (or infinite current spreading), the current is homogeneously spread out over the entire mesa area, and indeed, *J*_s,0_ approaches *J*_mesa_ according to eq 10. On the other hand, at infinite current (or infinitesimal current spreading), the current flows only downward and *J*_*s*,0_ approaches *J*_grid_. At any finite current, *J*_s,0_ approximates the current density experienced by the active LED area. In this way, *J*_s,0_ can be utilized to interpret the current–voltage characteristics of extended-perimeter LEDs, as will be demonstrated in the following.

### III.II Current–Voltage Characteristics

In the typical forward bias current density–voltage (J–V) characteristic of an LED, nonradiative recombination via defect levels (SRH recombination) with an ideality factor *n* = 2 dominates at low voltages, potentially transitioning to an *n* = 1 regime, where band-to-band radiative recombination dominates at higher voltages.^31,32^ Finally, above a certain voltage threshold, series resistance becomes the dominant mechanism, requiring increasingly large external voltages to further raise the current. This is observed as a downward bend of the *J*–*V* curve from the *n* = 1 regime. Figure 4a,b depicts the *J*_s,0_–*V* characteristics of LEDs with varying perimeter-to-contact distance (*L*) and a grid width (*W*) of 150 and 950 μm, respectively. The dashed lines indicate the slopes associated with the *n* = 2 and *n* = 1 regimes. Interestingly, the LEDs in this study operate predominantly in a regime between that of *n* = 2 and *n* = 1 at voltages below 1.1 V, indicating an intermediate recombination mechanism. At higher voltages, the series resistance losses start to dominate before the *J*–*V* curves fully transition to the *n* = 1 regime. To obtain a measure for the nonradiative recombination rate, the *n* = 2 dark saturation current densities (*J*_02_) were obtained by fitting the J–V curves to the single-diode equation *J*_mesa_ = *J*_02_ exp(*qV*/2*k*_B_*T*) at low currents, where *J*_s,0_ ≈ *J*_mesa_.

With a larger distance of the perimeter to the contact, the perimeter-to-surface area (P/A) ratio decreases, which results in a smaller relative influence of perimeter recombination. For *W* = 150 μm (Figure 4a), this effect is clearly observed as it causes the *J*–*V* curves to shift down with an order of magnitude drop in the *n* = 2 saturation current density from *J*_02_ = 5.2 × 10^–11^ to 5.5 × 10^–12^ A/cm^2^ if *L* is increased from 25 to 1000 μm. This indicates a considerably lower nonradiative recombination rate. By plotting the *J*_02_ vs the P/A ratio (see Figure S4), it is possible to resolve the overall contributions of both the bulk and perimeter to the nonradiative recombination according to^33^
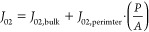
11

This yields the bulk recombination saturation current density *J*_02,bulk_ = (2.0 ± 1.4) × 10^–12^ A/cm^2^ and the linear recombination current density at the perimeter *J*_02,perimeter_ = (2.36 ± 0.13) × 10^–13^ A/cm (see Figure S4), which indicates a strong contribution of the perimeter to the total nonradiative recombination for the larger P/A ratios.^33^

As *W* is increased to 950 μm (Figure 4b), the effect of extending the perimeter is diminished since the smaller P/A ratio of larger LEDs already decreases perimeter recombination. This confirms that, indeed, perimeter extension and not the difference in mesa area causes the downshift of the *J*_s,0_–*V* curves seen in Figure 4a, as for *W* = 950 μm, the curves for different mesa areas do overlap below 1.1 V with an average *J*_02_ of (8.7 ± 2.7) × 10^–12^ A/cm^2^ (see also Figure S4).

As already stated above, before the *J*–*V* curves fully transition to the regime where radiative recombination dominates (only a slight increase in slope toward *n* = 1 can be seen at 1.2 V in Figure 4b and for *L* up to 100 μm in Figure 4a), series resistance causes all curves to bend down for voltages around 1.15–1.3 V and higher. Interestingly, for *W* = 150 μm (Figure 4a), the series resistance appears to increase (i.e., a higher voltage is required to reach the same *J*_s,0_) with increasing perimeter extension, while this is not the case for *W* = 950 μm (Figure 4b). The main contributors to the total series resistance in the discussed LEDs are vertical resistance in the semiconductor layers, metal–semiconductor contact resistance at the front and back of the LED, and resistance in the metal front contact grid and in the wire bonds. These resistances should all be constant with a constant grid width. In addition, the fact that the spread in the series resistance regime is not seen for *W* = 950 μm indicates that a potentially varying lateral resistance in the semiconductor layers with different mesa sizes also has negligible influence on the *J*–*V* characteristics.

**Figure 5:**
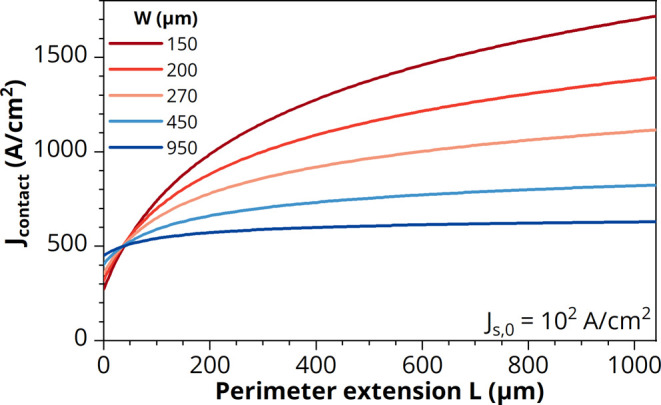
Current density through the interface between the front contact grid and the semiconductor (*J*_contact_) as a function of *L* at *J*_s,0_ = 10^2^ A/cm^2^ in GaAs/InGaP LEDs with a CSL and front contact grid width *W* = 150, 200, 270, 450, and 950 μm, where the contact area of the metal grid increases from 8.3 × 10^3^ to 2.0 × 10^5^ μm^2^ with increasing front contact grid width.

The cause for the observed shift to higher voltages with increasing perimeter extension in the series resistance regime of Figure 4a is therefore not due to a difference in series resistance but in fact due to a difference in required input current. In order to reach the same *J*_s,0_ in the LED, the input current and therefore the input voltage (with constant resistance) need to be higher if the perimeter extension is larger due to the dependence of *L*_s_ and *J*_s,0_ on *L* (eq 10). The increased input current with a larger perimeter extension enhances the resistive voltage losses, especially in the series resistance contributors located before the point where current density decreases due to current spreading. Specifically, these contributors include the wire bonds, metal grid, and front metal–semiconductor contact. As an example, Figure 5 illustrates the dependence of the current density through the interface between the front contact grid and the semiconductor (*J*_contact_ = *I*_input_/*c*_f_·*W*^2^, where *c*_f_ is the front contact grid coverage) on the perimeter extension *L* at constant *J*_s,0_. This example also clarifies why no difference in series resistance is observed between the devices in Figure 4b. With a larger front contact grid width (*W* = 950 μm) and therefore more contact area, the current density through the metal–semiconductor interface and the associated resistive losses remain fairly constant with *L* up to 1000 μm. In contrast, for *W* = 150 μm, *J*_contact_ increases rapidly with *L* such that for *L* > 100 μm, the voltage losses start affecting the series resistance regime and prevent the transition to the *n* = 1 regime (see Figure 4a).

In addition to the voltage losses due to increasing the perimeter extension, all *J*–*V* curves in Figure 4 display a similar downward bend due to a series resistance independent of perimeter extension. Using the methods developed by Algora et al.,^34^ we have identified the dominant contributors to the common series resistance in the LEDs in this work to be the vertical resistance in the substrate, the resistance in the wire bonds, and the front and back contact resistances. The former two contributions can, in principle, be eliminated by removing the substrate, yielding a thin-film LED device, and extending the contact pads outside of the mesa area to allow for direct probing. The latter contributions would require optimizations in the dopants, metal compositions, and annealing for decreased contact resistivity. Reducing these contributions would enable higher current densities in the *n* = 1 radiative recombination regime and allow for higher perimeter extensions to be used without significant voltage losses.

However, in the present LEDs, choosing the optimal perimeter extension remains a compromise between less nonradiative recombination but higher voltage losses with larger perimeter-to-contact distances. Moreover, the results (Figure 4) show that the goal of reducing perimeter recombination can be reached both by increasing the perimeter-to-contact distance and by increasing the front contact grid width, as both approaches result in a lower P/A ratio. However, in the context of demonstrating ELC, we are limited in grid width by the high current density needed in the LEDs (∼10^1^–10^2^ A/cm^2^).^15^ To this end, the extended-perimeter LEDs allow us to decrease the P/A ratio without affecting the maximum attainable current density in the active LED area under the front contact grid for a certain maximum current that the power supply and wire bonds can withstand. Thus, the grid width should be maximized according to the desired maximum current density, after which the perimeter can be extended to decrease the P/A ratio further, up to the point where voltage losses start to affect the performance.

**Figure 6:**
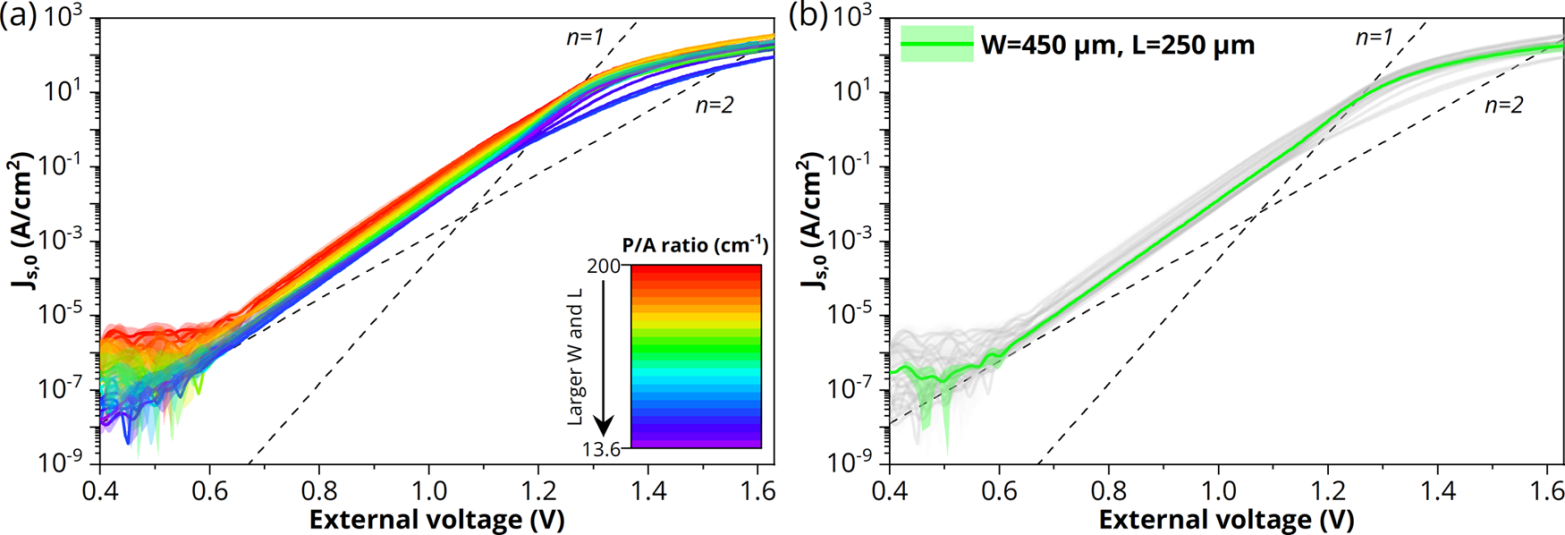
(a) Average current density–voltage (*J*_s,0_–*V*) characteristics in forward bias of GaAs/InGaP LEDs with a CSL and with varying front contact grid widths (*W* = 150, 200, 270, 450, 950 μm) and varying perimeter extensions (*L* = 25, 50, 100, 250, 500, 1000 μm) and all combination thereof. (b) The *J*_s,0_–*V* curve of (a) in which the combination*W* = 450 and *L* = 250 μm is highlighted. In both panels (a) and (b), the dashed lines represent the single-diode equation *J* = *J*_0*n*_ exp(*qV*/*nk*_B_*T*) with ideality factor *n* = 1 (*J*_01_ = 5.5 × 10^–21^ A/cm^2^) and *n* = 2 (*J*_02_ = 5.5 × 10^–12^ A/cm^2^).

As an example of applying these criteria, Figure 6a shows the *J*_s,0_–*V* curves of all fabricated LEDs in this study, both with varying front contact grid widths (*W* = 150–950 μm) and varying perimeter extensions (*L* = 25–1000 μm) and all combinations thereof. This clearly demonstrates the trend of a lower nonradiative recombination rate with a lower P/A ratio, i.e., higher *W* and/or *L*. Based on the criteria described above, the best compromise out of the fabricated devices is selected in Figure 6b, which corresponds to an LED with *W* = 450 μm—to yield high enough current density for reaching the QE maximum—and *L* = 250 μm, which prevents excessive voltage losses (see also Figure 5). This corresponds to the *J*–*V* curve with the lowest possible current density in the nonradiative recombination regime <1.1 V and the highest possible current density in the series resistance regime >1.3 V, in other words, the steepest slope (closest to *n* = 1) in-between these regimes. Based on eqs 5 and 10, the current spreading length in this LED operating around the maximum EQE can now be approximated to be 95 μm. This implies that the current density at the position of the perimeter (*x* = 250 μm in eq 4) is only a fraction 0.12 of the original *J*_s,0_ under the *W* × *W* grid width area.

### III.III Quantum Efficiency Optimum

**Figure 7:**
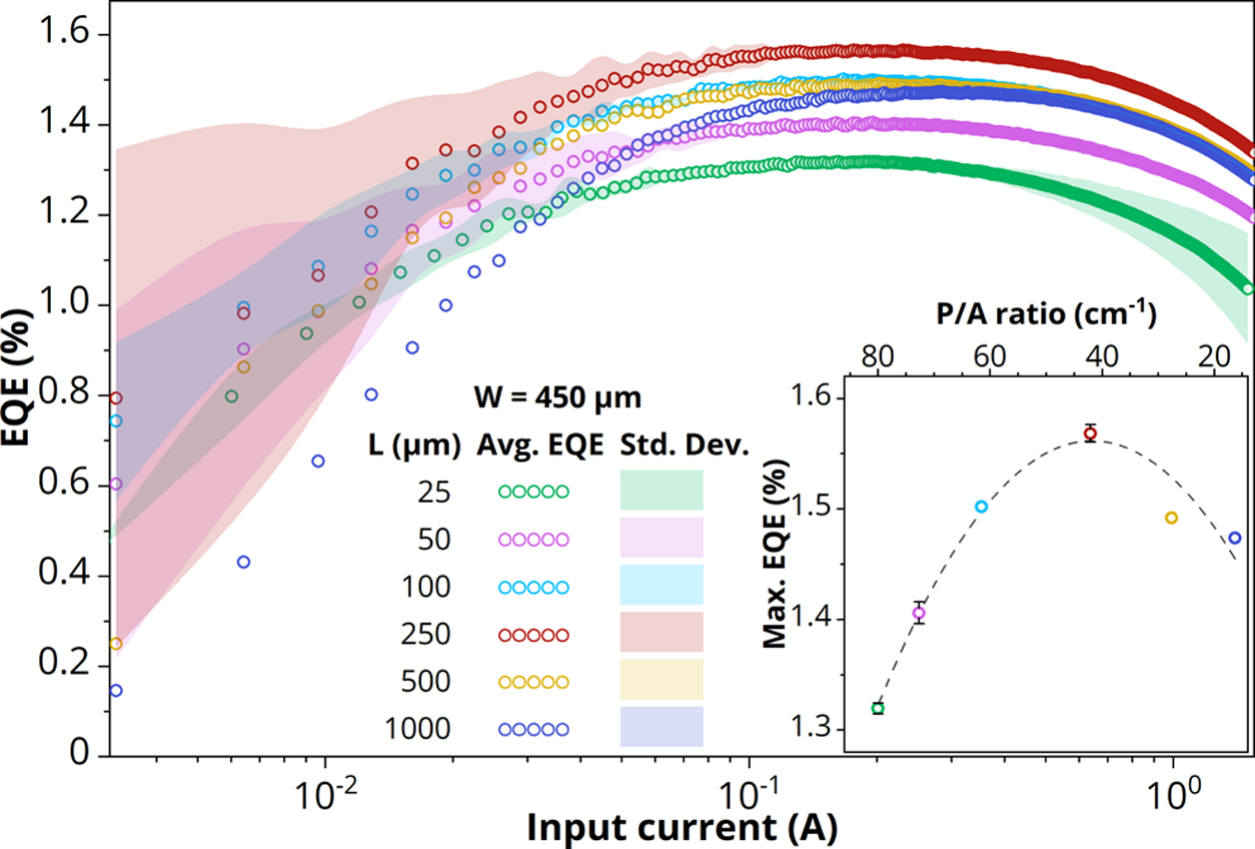
Average EQE as a function of input current for GaAs/InGaP LEDs with a CSL. The perimeter extension is varied from 25 to 1000 μm with a constant front contact grid (*W* = 450 μm). Inset shows the maximum EQE as a function of P/A ratio. The dashed line is a guide to the eye.

In order to confirm the findings from the *J*–*V* curves, Figure 7 depicts the measured external quantum efficiency as a function of the input current of LEDs with a 450 μm front contact grid width and varying perimeter extensions. Indeed, the maximum EQE increases relatively with 19% from *L* = 25 μm up to *L* = 250 μm before declining again (see the inset in Figure 7). This is in agreement with the decrease in nonradiative recombination and simultaneously the increase in resistive losses, with longer perimeter extensions, as observed from the *J*–*V* curves (Figure 4). Note that the absolute EQE values obtained in this study are low as a result of the followed research approach, which avoids potential variations in the LEE by processing and characterizing the different LED geometries on the growth substrate (see Section I).

Moreover, the competition between nonradiative defect-related Shockley–Read–Hall (SRH) recombination, radiative recombination, and Auger recombination results in the parabolic shape observed in each EQE vs *I* curve.^7,14^ This competition results in an EQE maximum at an input current of approximately 0.15 A, corresponding to 74 A/cm^2^ if one assumes that the current is confined to the *W* × *W* grid width area. Alternatively, when current spreading is taken into account, the current density through the junction underneath the *W* × *W* grid width area (*J*_s,0_) is 36 A/cm^2^, calculated using the model presented in this study (eq 10). The EL spectrum at this EQE maximum, taken at a corresponding external bias voltage of 1.37 V, has a weighted average photon energy of 1.44 eV. This confirms that, indeed, *hν*/*qV*_b_ > 1 (eq 2) at this operating point, which fulfills one of the criteria for demonstrating ELC. Further reduction of SRH recombination would shift the EQE maximum to even lower external bias voltages, which, in addition to decreasing series resistance, would further increase the voltage efficiency.

The measured maximum EQE ultimately is a product of the IQE and the LEE (see eq 3). To estimate the IQE—the primary focus of this work—from the EQE, a Photon Dynamics model developed by van Eerden et al. is used (see the Supporting Information for more details).^26^ Using this model, the probabilities of escape and reabsorption of the internal luminescence in the LED structure of Figure 1a can be simulated. Moreover, in order to correct for grid coverage in extended-perimeter LEDs, an estimate of the area where light is emitted is required. For this, an area of (*W* + 2·*L*_s_)^2^ is chosen, as most light will be emitted within one current spreading length from the front contact grid. Under these assumptions, the LED with the best obtained EQE (1.57% for *W* = 450 μm and *L* = 250 μm, see Figure 7) yields an IQE of approximately 89%. Compared to the LED with tight perimeter extension (*W* = 450 μm and *L* = 25 μm, IQE = 85%), this corresponds to a 5% relative increase in IQE, achieved by simply extending the perimeter-to-contact distance farther than the lateral current spreading length. This improvement is an important first step toward the demonstration of ELC. Further developments to ultimately achieve net cooling at practical cooling powers, i.e., an above unity WPE at voltages close to the band gap energy, require the combination of these results with state-of-the-art approaches to enhance the LEE. Without an optimized light extraction scheme, the majority of photons are lost within the device as heat, therefore competing with cooling. Additionally, measurements of the IQE using photoexcitation on GaAs/InGaP heterostructures show that there is also still room for improvement in the IQE.^12,35,36^ This can be addressed by optimizing the internal LED epilayer structure and minimizing the series resistance to complement the extended perimeter and further approach unity IQE.

## IV Conclusions

In the perspective of utilizing light-emitting diodes for demonstrating electroluminescent cooling, a fabrication method is presented that enables a reduction in nonradiative perimeter recombination by decreasing the perimeter-to-surface area ratio. This constitutes extending the distance from the current injection point to the perimeter farther than current can spread laterally at the operating current density. Contrary to decreasing the perimeter-to-surface area ratio by simply increasing the front contact grid size, the present approach maintains a small active LED area under the front contact grid and, consequently, a high maximum attainable current density. As a first-order approximation, the well-known 1-D current distribution relation for stripe-shaped contacts is expanded to 2-D to determine the level of lateral current spreading in the extended-perimeter LEDs, which was experimentally observed in confocal electroluminescence scanning microscopy maps of p–i–n GaAs/InGaP double heterojunction LEDs. The calculations aid in the interpretation of the LED current–voltage characteristics, which reveal a significant drop in nonradiative recombination rates with larger perimeter extension, as evidenced by an order of magnitude decrease in the *n* = 2 saturation current density. This is ascribed to a reduction in perimeter recombination due to a strongly reduced current density at the perimeter.

Series resistance losses, requiring increasing voltages to sustain the same current density in the LED, are found to be the limiting factor for the maximum perimeter extension. On the other hand, the size of the active LED area under the front contact grid is limited by the desired maximum current density. For demonstrating electroluminescent cooling in LEDs, where high current densities are required to achieve the desired cooling power density, the optimal device dimensions of the fabricated LEDs are therefore found to be a front contact grid width of 450 μm and a perimeter-to-contact distance of 250 μm. This yields a 19% relative increase in external quantum efficiency compared to a tight perimeter extension of 25 μm. Utilizing an in-house developed Photon Dynamics model, the corresponding relative increase in internal quantum efficiency is estimated to be 5%.

This significant improvement in internal quantum efficiency presents an important step toward demonstrating electroluminescent cooling in LEDs and additionally contributes to the advancement of semiconductor devices in general by addressing the limitations imposed by perimeter recombination. To ultimately achieve net cooling at practical cooling powers, the results of this study will have to be combined with state-of-the-art approaches to maximize light extraction, and further improvements of IQE by optimizing the internal LED epilayer structure and minimizing the series resistance.

## Supporting Information Available

It describes the required correction to the EL photon count from confocal EL scanning microscopy to be directly proportional to the current distribution. It also shows the confocal EL scanning microscopy data before correction and the homogeneity of current between gridlines with and without CSL. Moreover, the *J*_02_ vs the P/A ratio is plotted to extract the overall contributions of both the bulk and perimeter to the nonradiative recombination. Lastly, the Photon Dynamics model used to estimate the IQE is explained in detail (PDF)
