# Supplementary material for: Reduced Surface Recombination in Extended-Perimeter LEDs toward Electroluminescent Cooling
Source: ACS Appl Electron Mater. 2024 Feb 13;6(2):1483–92. doi: 10.1021/acsaelm.3c01816 (PMC10902842; doi:10.1021/acsaelm.3c01816)
Supplement: Supplementary file 1 — el3c01816_si_001.pdf [file el3c01816_si_001.pdf]

Supporting Information

Reduced Surface Recombination in Extended-Perimeter LEDs towards Electroluminescent Cooling

Luc M. van der Krabben[a*], Natasha Gruginskie[a], Maarten van Eerden[a], Jasper van Gastel[a], Peter Mulder[a], Gerard J. Bauhuis[a], Dinar Khusyainov[a], Dima Afanasiev[a], Elias Vlieg[a], John J. Schermer[a]

[a]Radboud University, Institute for Molecules and Materials, 6525 AJ Nijmegen, The Netherlands

*Corresponding author. E-mail: luc.vanderkrabben@ru.nl

EL microscopy

In confocal electroluminescence (EL) scanning microscopy the collected data is a photon count, related to the EL emission intensity. However, the metric of interest for assaying the current spreading model is the current distribution. To account for the non-linear relation between the intensity of spontaneous emission and current, the EL microscopy data requires correction to be directly proportional to the current distribution.[1] The intensity of spontaneous emission P_s is described by

$$P_s = P_{s,0} \exp\left(\frac{V_j}{V_T}\right) \rightarrow \frac{P_s}{P_{s,0}} = \exp\left(\frac{V_j}{V_T}\right) \rightarrow \ln\left(\frac{P_s}{P_{s,0}}\right) = \frac{V_j}{V_T} \tag{S1}$$

where V_j is the junction voltage and V_T is the thermal voltage ($k_B T/e$). k_B, T and e are the Boltzmann constant, temperature, and elementary charge, respectively. Similarly, the current through a diode can be written as

$$I = I_0 \exp\left(\frac{V_j}{\eta V_T}\right) \rightarrow \frac{I}{I_0} = \exp\left(\frac{V_j}{\eta V_T}\right) \rightarrow \ln\left(\frac{I}{I_0}\right) = \frac{V_j}{\eta V_T} \rightarrow \frac{V_j}{V_T} = \eta \ln\left(\frac{I}{I_0}\right) \tag{S2}$$

where η is an ideality factor. Equating V_j/V_T in both equations yields

$$\ln\left(\frac{P_s}{P_{s,0}}\right) = \eta \ln\left(\frac{I}{I_0}\right) \tag{S3}$$

Therefore, plotting $\ln\left(\frac{P_s}{P_{s,0}}\right)$ vs. $\ln\left(\frac{I}{I_0}\right)$ yields η as the slope. $P_{s,0}$ and I_0 are constants set to 1 mW and 1 mA, respectively, to cancel out the units, as we are only regarding the relative slope. This is depicted in Fig. S1, where the absolute emission power is acquired with an Avantes Starline AvaSpec-ULS2048CL-EVO-RS spectrometer with 100 µm slit size, connected to an integrating sphere. The injection current is supplied by a Keithley 2460 SourceMeter. The EL signal is corrected for the measurement distance to the integrating sphere. From this, the correction factor η can be determined for a specific current (see Fig. S1c), which is used to correct the emission intensity data to be proportional to current, according to

$$\frac{I}{I_0} = \left(\frac{P_s}{P_{s,0}}\right)^{\frac{1}{\eta}} \tag{S4}$$

The EL microscopy data of p-i-n DH GaAs/InGaP LEDs (W = 950 µm and L = 1000 µm) with and without InGaP-Si CSL before the above-described correction are shown in Fig. S2 and Fig. S3. These figures are respectively acquired in the central region of the front contact edge (just aside from the contact pads for wire bonding) and in the center of the LED between gridlines.

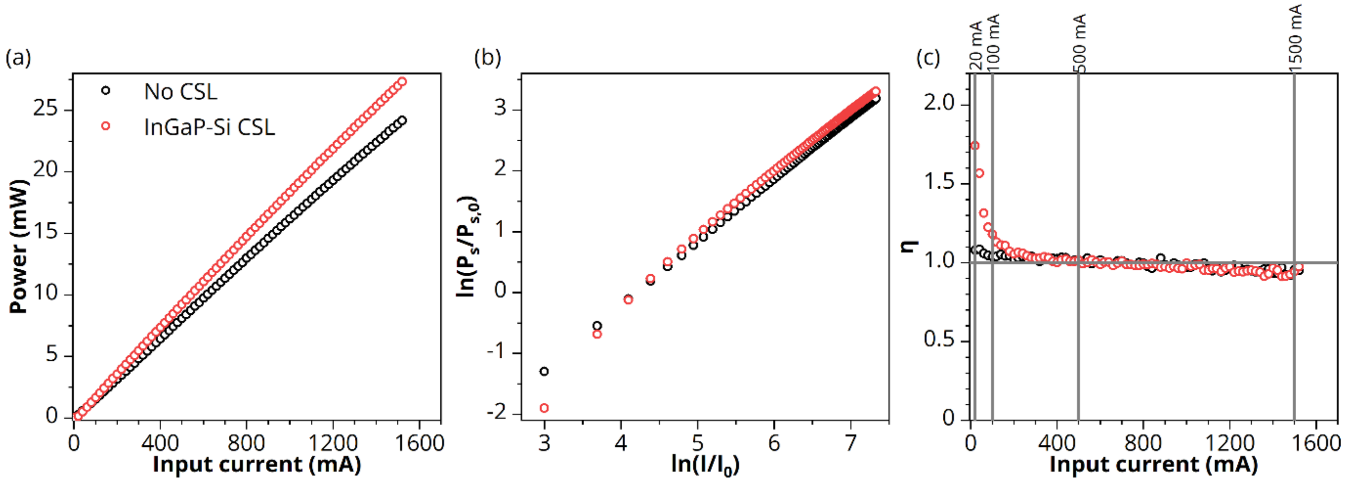

Fig. S1: ***(a)*** *Electroluminescence emission power vs. input current for GaAs/InGaP LEDs (W = 950 μm and L = 1000 μm), with (red) and without (black) InGaP-Si CSL.* ***(b)*** *The data plotted as* $\ln\left(\frac{P}{1\ mW}\right)$ *vs.* $\ln\left(\frac{I}{1\ mA}\right)$*.* ***(c)*** *The slope of (b), η, vs input current. Reference lines are added for the currents at which EL microscopy images are acquired.*

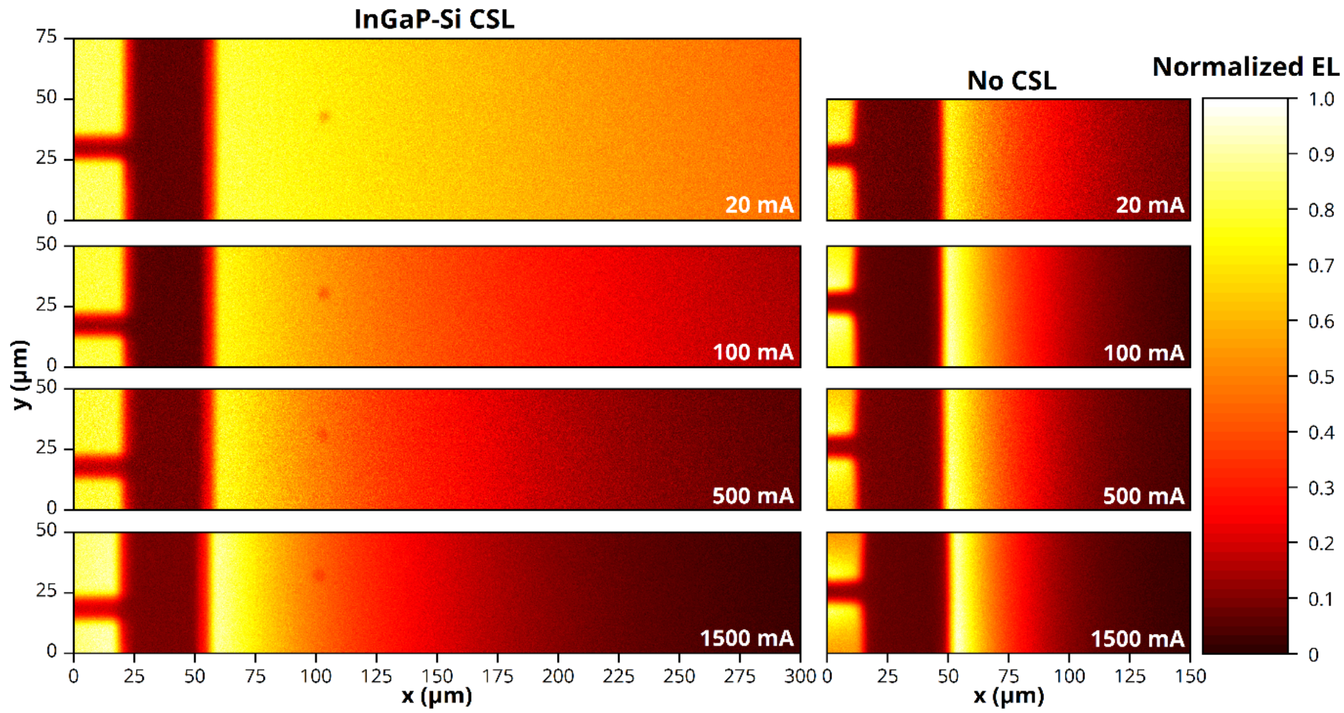

Fig. S2: *Confocal EL scanning microscopy photon count maps (averaged from 3 maps, normalized on maximum intensity at x≈55 μm) of GaAs/InGaP LEDs (W = 950 μm and L = 1000 μm) with and without InGaP-Si CSL and input currents of 20, 100, 500 and 1500 mA. The images were taken from the central region of the front contact edge, just aside from the contact pads for wire bonding. The data is before correction to be proportional to current.*

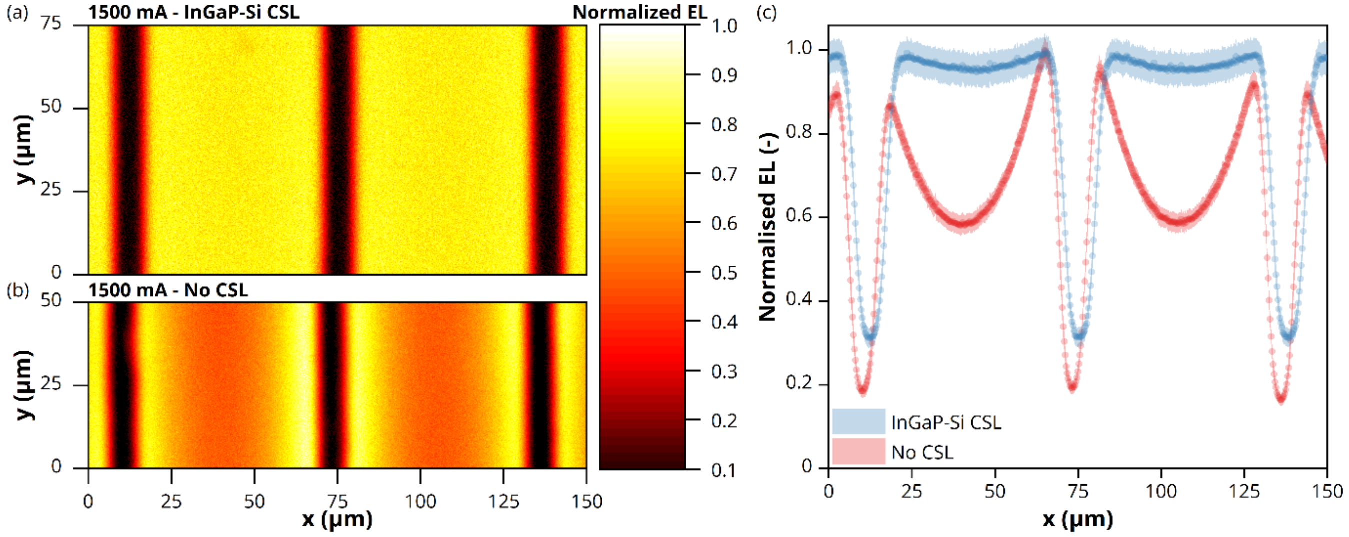

Fig. S3: ***(a-b)*** *Confocal EL scanning microscopy photon count maps (averaged from 3 maps, normalized on maximum intensity at $x \approx 65$ µm) of GaAs/InGaP LEDs (W = 950 µm and L = 1000 µm) with (a) and without (b) InGaP-Si CSL imaged between gridlines at the center of the LED at an input current of 1500 mA. This shows the homogeneity of EL signal between gridlines with CSL.* ***(c)*** *Normalized EL as a function of x, as deduced from the EL photon count maps in (a) and (b) by averaging over the values along the y-direction (the data points represent the average value and the shaded region the standard deviation). The data is before correction to be proportional to current.*

Bulk and Perimeter recombination

Fig. S4 shows the n=2 dark saturation current density (J_{02}) plotted versus the perimeter-to-surface area (P/A) ratio of all LEDs produced in this study. The J_{02} values are obtained by fitting the single diode equation

$$J_{mesa} = J_{02} \exp\left(\frac{qV}{2k_BT}\right) \quad \text{(S5)}$$

with ideality factor n=2, to the low voltage regime of the J_{mesa}-V curves of the fabricated devices.[2] According to

$$J_{02} = J_{02,bulk} + J_{02,perimter} \cdot \left(\frac{P}{A}\right) \quad \text{(S6)}$$

a linear fit through the data of Fig. S4 yields the bulk recombination current density $J_{02,bulk}$ and the linear recombination current density at the perimeter $J_{02,perimeter}$.[2]

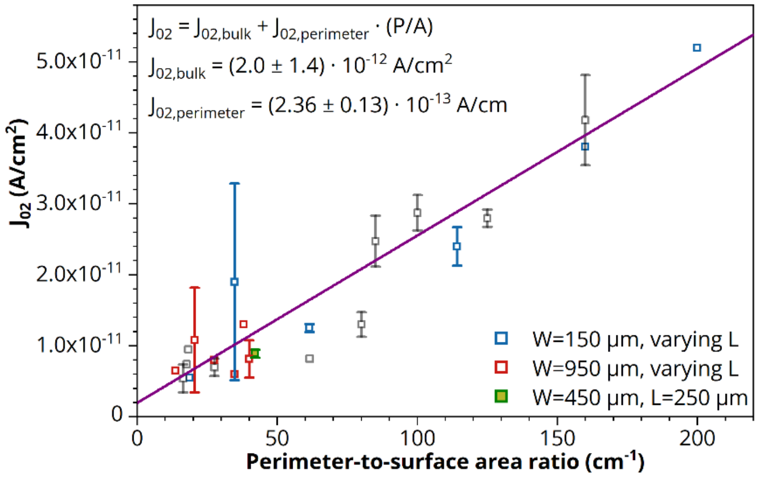

Fig. S4: *Dark saturation current density with ideality factor n=2 (J_{02}) vs. the perimeter-to-surface area ratio (P/A) of all fabricated GaAs/InGaP LEDs in this study with varying front contact grid width W and perimeter extension L (open squares). The LEDs with W=150 µm are marked in blue, while the LEDs with W=950 µm are marked in red. The LED with W=450 µm and L=250 µm is marked in green. The grey markers represent LEDs with other values of W and L. The purple line represents the best fit to Eq. S6.*

Estimation of LED IQE

The external quantum efficiency (EQE) of an LED is the product of the internal quantum efficiency (IQE) and the light extraction efficiency (LEE):

$$EQE = IQE \cdot LEE \tag{S7}$$

A common approach in photovoltaics is to describe the LEE in terms of the probability that an internally generated photon directly escapes through the front side of the device (P_{esc}) and the probability that it is reabsorbed in the active layer (P_{abs}):[3–5]

$$LEE = \frac{P_{esc}}{1 - P_{abs} \cdot IQE} \tag{S8}$$

Reabsorption in the active layer can lead to the radiative emission of a new photon with probability IQE (photon recycling), which again has a probability of P_{esc} to contribute to the LEE. The IQE, defined as the ratio of internally generated photons to injected charge carriers, can then be written as

$$IQE = \frac{EQE}{P_{esc} + EQE \cdot P_{abs}} \tag{S9}$$

In order to estimate the IQE of the LEDs with the lowest and highest EQE (L=25 and 250 µm, respectively, with W=450 µm, see Fig. 7 in the main text), assuming no injection losses, P_{esc} and P_{abs} are calculated using an in-house developed Photon Dynamics model.[4] In short, the model considers isotropic and homogeneous internal emission of light intensity in the active layer, distributed in photon energy E according to the Van Roosbroeck-Shockley relation[6]

$$S_{int}(E) = 2\frac{\alpha(E)n^2(E)E^2}{h^3c^2}\frac{1}{\exp\left(\frac{E}{k_BT}\right) - 1} \tag{S10}$$

where α is the GaAs absorption coefficient, n is the refractive index, h is the Planck constant, c is the speed of light in vacuum, k_B is the Boltzmann constant and T is the temperature. Intensity is initialized and traced as it propagates through the active layer in a position-, energy- and angle-dependent manner. It is diminished by the loss processes of Beer-Lambert absorption in the active layer and transmission (non-reflection) at both interfaces, until the remaining intensity falls below the threshold value of 10^{-7} of the initialized intensity. Intensity that is reabsorbed in the active layer is assigned to P_{abs}, intensity that is transmitted at the front interface is considered to couple out and is assigned to P_{esc}. Finally, intensity 'transmitted' at the rear interface is assigned to the probability of parasitic absorption P_{par}. This procedure yields energy- and angle-dependent spectra of P_{abs}, P_{esc} and P_{par}, which are integrated with respect to energy and angle to arrive at single probability values. The external electroluminescence spectrum is given by the product of $S_{int}(E)$ and $P_{esc}(E)$. The reflection and transmission coefficients are calculated using a transfer-matrix formalism that considers the thicknesses and complex refractive indices of the front and rear layer stacks, that clad the active layer (see Fig. 1 in the main text for the layer structure). Reflection is considered to be specular in all cases.

The spectral shape of the measured EL spectra of the LEDs with the lowest and highest EQE (L=25 and 250 µm, respectively, with W=450 µm, see Fig. 7 in the main text) is compared to the spectrum predicted by the Photon Dynamics model in Fig. S5. Overall, the agreement is satisfactory, except for an extended sub-bandgap tail in the measured spectra that is not reproduced by the model, and a slight deviation on the high energy side of the L=25 µm spectrum. The latter could be due to a higher junction temperature during the measurements of this spectrum, since it was acquired at higher current density. The former issue is related to band tailing, for which the model accounts in a phenomenological manner, by modeling the GaAs absorption coefficient using $\alpha \sim \alpha_0\sqrt{E - E_g}$, where α_0 is the fit to the GaAs absorption coefficient from literature[7] and E_g is the bandgap energy, which is then convoluted using the exponential $\alpha \sim \exp\left(\frac{E-E_g}{E_0}\right)$, where E_0 is the Urbach energy. The modeled spectrum in Fig. S5 was calculated using an Urbach energy of 7 meV for the GaAs active layer, a typical value for intrinsic GaAs.[8]

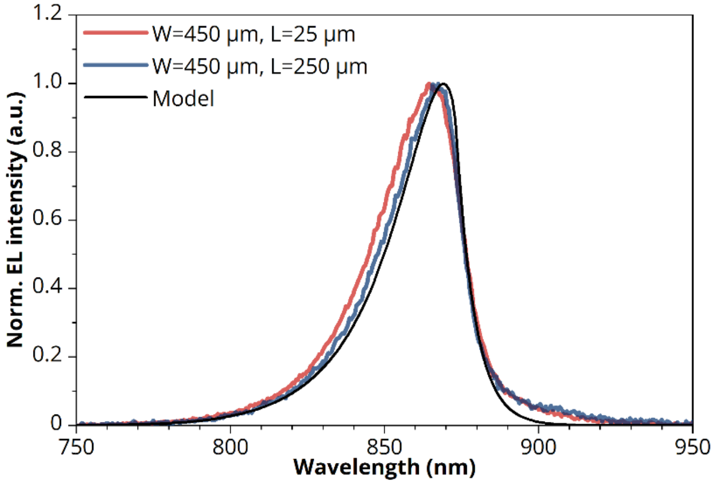

Fig. S5: *Comparison of the modeled EL spectrum with measured spectra of the GaAs/InGaP LEDs with the lowest and highest EQE (W=450 µm, L=25 and W=450 µm, L=250 µm, respectively, see Fig. 7 in the main text). The modeled spectrum was calculated assuming the layer structure shown in Fig. 1 in the main text, an Urbach energy of 7 meV for the GaAs active layer and a temperature of 300 K.*

In order to determine global P_{abs} and P_{esc} values for the LEDs used in this study, they are calculated for a structure without front side metallization and a structure with a fully metallized front side (100% coverage). The values are subsequently weighted according to the actual front contact grid coverage. To determine the grid coverage of an extended perimeter LED, in which light emission drops off approximately exponentially with distance from the front contact grid (see Eq. 4 in the main text), the total area where light is visibly emitted is estimated conservatively as $(W + 2 \cdot L_s)^2$, with W the front contact grid width and L_s the current spreading length. A lower estimate of this area would result in a higher coverage and, consequently, a lower global P_{esc} value and a higher estimate of IQE from the measured EQE value (Eq. S9). Table S1 presents an overview of the modeled probabilities of reabsorption and escape, the measured EQE values and the derived LEE and IQE. The low value of P_{esc} (1.18%) is caused by the narrow escape cone of GaAs ($\frac{1}{4n^2} \approx 1.9\%$) and reabsorption of part of the intensity emitted within the escape cone. Roughly 46% of the internal emission is reabsorbed in the active layer, while the remaining emission is lost to the substrate. Using these probabilities, LEE values of 1.77% (1.56%) are calculated for LEDs with the highest (lowest) EQE (L=25 and 250 µm with W=450 µm, see Fig. 7 in the main text), yielding IQE values of 89% (85%).

Table S1: *Overview of modeled probabilities of reabsorption in the active layer (P_{abs}) and escape (P_{esc}) for the fabricated GaAs/InGaP LEDs, both with and without full front-side metallization.*

	P_{abs} (%)	P_{esc} (%)	EQE (%)	LEE (%)	IQE (%)
With full front-side metallization	46.0	0	-	-	-
Without front-side metallization	46.3	1.18	-	-	-
Global (W=450 µm, L=250 µm, est. cov.: 12.2%)[a]	46.2	1.04	1.57	1.77	89
Global (W=450 µm, L=25 µm, est. cov.: 20.1%)[a]	46.2	0.95	1.32	1.56	85

[a]The measured EQE is shown for the LEDs with the highest (W=450 µm, L=250 µm) and lowest (W=450 µm, L=25 µm) measured EQE values, for which the derived LEE and IQE are provided.

Error analysis of LED IQE

The main sources of error for the estimation of the IQE are the assumptions made in the Photon Dynamics model (e.g., the partial use of ray optics), the grid coverage estimation and non-idealities in the fabricated LEDs. The impact of the latter can be estimated by calculating P_{abs} and P_{esc} for a range of input parameters for which uncertainty exists. Examples are the active layer thickness d, the junction temperature T and the Urbach energy E_0. Therefore, we did simulations for these parameters in the limits d=285-315 nm, T=300-340 K and E_0=5-9 meV and show the extreme values of P_{abs} and P_{esc} in Table S2. The probabilities for T=340 K are all in between those shown in Table S1. The minimum and maximum IQE of the LED with the best EQE (W=450 µm, L=250 µm) derived in this way are 88% and 90%, respectively. The reason for the small variation in IQE over the uncertainty range of the input parameters is that they have opposing effects on P_{abs} and P_{esc}, which counteracts their impact on the IQE.

Table S2: *Overview of the extreme values with varying input parameters of modeled probabilities of reabsorption in the active layer (P_{abs}) and escape (P_{esc}) for the fabricated GaAs/InGaP LEDs, both with and without full front-side metallization.*

	P_{abs} (%)	P_{esc} (%)	EQE (%)	LEE (%)	IQE (%)
d=315 nm, E_0=5 meV, T=300 K					
With full front-side metallization	47.9	0	-	-	-
Without front-side metallization	48.1	1.17	-	-	-
Global (W=450 µm, L=250 µm, est. cov.: 12.2%)[a]	48.1	1.03	1.57	1.78	88
d=285 nm, E_0=9 meV, T=300 K					
With full front-side metallization	43.9	0	-	-	-
Without front-side metallization	44.1	1.20	-	-	-
Global (W=450 µm, L=250 µm, est. cov.: 12.2%)[a]	44.1	1.05	1.57	1.75	90

[a]*The measured EQE is shown for the LEDs with the highest (W=450 µm, L=250 µm) measured EQE values, for which the derived LEE and IQE are provided.*

The error as a result of the assumptions in the Photon Dynamics model itself is harder to estimate. However, by varying both P_{esc} and P_{abs} by ±5%$_{rel}$ the sensitivity of the IQE to systematic errors in the model can be gauged. This leads to an IQE range for the best LED between 86% and 95%, when both P_{esc} and P_{abs} are varied by +5% or by -5%, respectively. Note that this uncertainty does not affect the IQE comparison between the LEDs with highest and lowest measured EQE, since these calculations use the same probabilities, except for a different grid coverage weighting. The error resulting from the grid coverage estimation of the best LED, having an extended perimeter, can be estimated by assuming the grid coverages of the LEDs with L=25 µm and L=250 µm to be equal (20.1%). The IQE of the best LED would then be 94%, instead of 89%. Naturally, the IQE of the LED with the lowest measured EQE would still be 85%.

References

(1) Sommers, H. S.; North, D. O. Experimental and Theoretical Study of the Spatial Variation of Junction Voltage and Current Distribution in Narrow Stripe Injection Lasers. *J. Appl. Phys.* **1977**, *48* (11), 4460–4467.

(2) Espinet-González, P.; Rey-Stolle, I.; Ochoa, M.; Algora, C.; García, I.; Barrigõn, E. Analysis of Perimeter Recombination in the Subcells of GaInP/GaAs/Ge Triple-Junction Solar Cells. *Prog. Photovoltaics Res. Appl.* **2015**, *23* (7), 874–882.

(3) Steiner, M. A.; Geisz, J. F.; Garcia, I.; Friedman, D. J.; Duda, A.; Olavarria, W. J.; Young, M.; Kuciauskas, D.; Kurtz, S. R. Effects of Internal Luminescence and Internal Optics on Voc and Jsc of III-V Solar Cells. *IEEE J. Photovoltaics* **2013**, *3* (4), 1437–1442.

(4) van Eerden, M.; van Gastel, J.; Bauhuis, G. J.; Vlieg, E.; Schermer, J. J. Comprehensive Analysis of Photon Dynamics in Thin-Film GaAs Solar Cells with Planar and Textured Rear Mirrors. *Sol. Energy Mater. Sol. Cells* **2022**, *244*, 111708.

(5) Rau, U.; Paetzold, U. W.; Kirchartz, T. Thermodynamics of Light Management in Photovoltaic Devices. *Phys. Rev. B - Condens. Matter Mater. Phys.* **2014**, *90* (3), 035211.

(6) Van Roosbroeck, W.; Shockley, W. Photon-Radiative Recombination of Electrons and Holes in Germanium. *Phys. Rev.* **1954**, *94* (6), 1558–1560.

(7) Sopra S.A. Company. Optical Data from Sopra SA. 2008.

(8) Johnson, S. R.; Tiedje, T. Temperature Dependence of the Urbach Edge in GaAs. *J. Appl. Phys.* **1995**, *78* (9), 5609–5613.
